# Pregnancy outcomes in women receiving growth hormone replacement therapy enrolled in the NordiNet® International Outcome Study (IOS) and the American Norditropin® Studies: Web-Enabled Research (ANSWER) Program

**DOI:** 10.1007/s11102-021-01138-3

**Published:** 2021-03-12

**Authors:** Beverly M. K. Biller, Charlotte Höybye, Paul Carroll, Murray B. Gordon, Anna Camilla Birkegård, Nicky Kelepouris, Navid Nedjatian, Matthias M. Weber

**Affiliations:** 1 Neuroendocrine Unit, Massachusetts General Hospital, Bulfinch 457B, Fruit St., Boston, MA 02114 USA; 2 Department of Endocrinology, and Department of Molecular Medicine and Surgery, Karolinska University Hospital and Karolinska Institute, Stockholm, Sweden; 3 Department of Endocrinology, Guy’s & St. Thomas’ NHS Foundation Trust, London, UK; 4 Allegheny Neuroendocrinology Center, Division of Endocrinology, Allegheny General Hospital, Pittsburgh, PA USA; 5 Epidemiology, Novo Nordisk A/S, Søborg, Denmark; 6 Novo Nordisk Inc., Plainsboro, NJ USA; 7 Global Medical Affairs – Rare Endocrine Disorders, Novo Nordisk Health Care AG, Zurich, Switzerland; 8 Unit of Endocrinology, Universitätsmedizin der Johannes Gutenberg-Universität Mainz, Mainz, Germany

**Keywords:** Human growth hormone, Adult growth hormone deficiency, IGF-I, Pregnancy, Outcome

## Abstract

**Purpose:**

Data on the safety of growth hormone (GH) replacement therapy during pregnancy are limited. We report a combined analysis of data from pregnant women treated with GH while enrolled in two non-interventional, multicenter studies: NordiNet® International Outcome Study (IOS) and the American Norditropin® Studies: Web-Enabled Research (ANSWER) Program.

**Methods:**

Pregnancy data were pooled from NordiNet® IOS and the ANSWER Program. Data were collected during routine clinic visits by participating physicians using a web-based system. Patients exposed to GH replacement therapy during pregnancy were included in the analysis.

**Results:**

The study population included 40 female patients with typical causes of adult GH deficiency (GHD). Overall, there were 54 pregnancies. Of these, 47 were exposed to GH between conception and delivery. In 48.9% of pregnancies exposed to GH, the dose was > 0.6 mg/day. GH was continued past conception and then stopped during the first, second, and third trimester, in 27.7%, 17.0%, and 2.1% of pregnancies, respectively. In 29.8%, GH was continued throughout pregnancy, with an unchanged dose in most cases. Of the 47 GH-exposed pregnancies, 37 (78.7%) progressed to normal delivery. There were three adverse events reported in two pregnancies.

**Conclusion:**

These real-world data suggest that there were no new safety signals related to GH exposure in women with GHD during pregnancy. These results are consistent with findings from previous studies reporting data in pregnancies exposed to GH at conception or throughout pregnancy. This observational study in additional pregnancies provides further evidence that GH exposure does not adversely affect pregnancy outcome.

**Clinical trial registration:** ClinicalTrials.gov NCT00960128 (date of registration: August 13, 2009) and NCT01009905 (date of registration: November 5, 2009).

## Introduction

Growth hormone (GH) deficiency (GHD) in adults is characterized by metabolic abnormalities (e.g., abdominal obesity, insulin resistance, reduced lean body mass), impaired psychosocial function, high levels of circulating cardiovascular risk biomarkers (C-reactive protein, plasminogen activator inhibitor [PAI-1], total cholesterol, low-density lipoprotein cholesterol), and fatigue [1–4]. GHD in adults can either continue from childhood, or be acquired in adulthood as a sequela of pituitary adenomas or their treatment [5, 6]. GHD may also arise from traumatic brain injury, or other pituitary or hypothalamic disorders [7]. Left untreated, GHD in adults can lead to premature morbidity and mortality [2, 8].

The goal of GH replacement therapy (GHRT) in adults with GHD is to normalize insulin-like growth factor-1 (IGF-I) levels, and thereby improve body composition, mitigate cardiovascular risk, maintain skeletal mass, and optimize physical and psychological function [9]. During pregnancy, GH is secreted from the placenta and the levels of placental GH increase throughout pregnancy from as early as 8 weeks, peaking around weeks 35–36 of gestation. The increase in placental GH levels during pregnancy in women with GHD from a pituitary condition is similar to that observed in individuals without GHD. Furthermore, the increase in placental GH levels is not suppressed by concomitant GHRT [10]. During this time, pituitary GH secretion decreases gradually to undetectable levels in maternal serum by week 36 of gestation [11].

GHRT is not approved for use during pregnancy. However, a number of women do conceive while receiving GHRT, either spontaneously (if pituitary function is retained) or during assisted conception treatment [12]. Data from KIMS (Pfizer International Metabolic Database) indicate that GHRT was partially continued or continued throughout the entire pregnancy in over half of the pregnancies reported in the study [13]. Although GHRT is not approved for use in pregnancy, in real-world clinical practice, some clinicians use GH replacement regimens in pregnant women with GHD, aiming to mirror the physiology of GH/IGF-I concentrations observed during pregnancy in healthy women. Thus, GHRT is continued during the first trimester, with the dose gradually reduced during the second trimester, and stopped altogether at the start of the third trimester [14].

Currently, there are no randomized controlled trials on the effect of GHRT during pregnancy. However, pregnancy outcomes after GHRT have been reported in the literature. In a case study, Muller et al. [15] described a 25-year-old woman with idiopathic isolated GHD who was treated with GH as part of a clinical trial. The patient became pregnant during the trial and was left on treatment until GH production from the placenta was evident. In this pregnancy, fetal size increase, birth weight (3.6 kg), and length (52 cm) were normal and no adverse events (AEs) were recorded.

Sakai et al. [16] reported a case of a Japanese patient initially diagnosed with pituitary dwarfism at 9 years of age. She received GHRT until she was 15 years old. Treatment was restarted when the patient was 24 years old following symptoms relating to GHD. The patient was found to be 8 weeks pregnant at age 28 years 7 months, at which point GHRT was stopped. The patient delivered a healthy girl at 40 weeks of pregnancy. No AEs were observed in either mother or baby. Two successful pregnancies and deliveries (at 38 and 40 weeks, respectively) have been described in another Japanese patient with evolving hypopituitarism (owing to pituitary stalk transection syndrome) who partially continued GHRT during pregnancy [17, 18]. In the second pregnancy, GHRT was continued until the end of the second trimester without any complications [18].

Wiren et al. [19] reported no major side effects nor a negative impact on maternal nor fetal outcomes in eight hypopituitary women who received GHRT during pregnancy. The patients received the same pre-gestational GH dose during the first trimester, with a gradual decrease of the dose during the second trimester, and discontinued treatment at the beginning of the third trimester.

In a study of 201 pregnancies in patients with GHD, there was no correlation between GHRT during pregnancy and pregnancy outcomes. Overall, 62% of the pregnancies exposed to GH (107/173) resulted in normal delivery with no birth defects [13]. A systematic literature review of fertility and pregnancy in women with hypopituitarism reported a live birth rate of 61–100% in those achieving pregnancy [20]. Neither GH replacement at conception nor during pregnancy were linked to pregnancy complications.

Despite these reports, more data on the safety of GHRT during pregnancy are needed. Therefore additional evidence from real-world data is highly relevant. In this paper, we report a combined analysis of data from pregnant women treated with GH while enrolled in two non-interventional, multicenter studies: the NordiNet® International Outcome Study (IOS) and the American Norditropin® Studies: Web-Enabled Research (ANSWER) Program [21, 22].

## Methods

### Study design

The study designs of NordiNet® IOS and ANSWER have been reported in detail in earlier reports [21, 22]. In summary, NordiNet® IOS (NCT00960128) and the ANSWER Program (NCT01009905) were non-interventional, multicenter registry studies monitoring the long-term outcomes of GHRT (with Norditropin®; Novo Nordisk A/S, Copenhagen, Denmark) in children and adults in clinical practice. NordiNet® IOS was ongoing between April 2006 and December 2016 and involved 469 clinics in 22 countries throughout Europe and the Middle East (Belgium, the Czech Republic, Denmark, Finland, France, Germany, Hungary, Ireland, Israel, Italy, Lithuania, Luxembourg, Montenegro, the Netherlands, Norway, Russia, Serbia, Slovenia, Spain, Sweden, Switzerland, and the UK). The ANSWER Program took place from June 2002 to September 2016 in 207 clinics in the USA. The two studies were complementary, with similar aims and using the same electronic data-management platform. Both studies were conducted with approval from relevant ethics committees, written consent from patients, and pseudonymization of all data in accordance with the Declaration of Helsinki, Guideline for Good Pharmacoepidemiology Practices, and regulatory requirements.

### Patient population

The study population included female patients who were exposed to GHRT before and during pregnancy in the course of real-world practice.

### Data collection

Data were collected for: pregnancies exposed to GH, GH exposure by country, baseline characteristics of exposed pregnancies, GHRT in exposed pregnancies, and the outcomes in the exposed and non-exposed pregnancies by age at conception.

All data were collected in accordance with routine medical practice and country-specific rules, and the study case-report forms allowed for all data collected including effectiveness endpoints to be adapted and edited according to local situations and practices. Therefore, because of differences between countries in the required fields for clinical findings, data were missing for some baseline variables. The conception date was computed as having occurred 38 weeks prior to either the actual delivery date or expected delivery date, as reported by the treating physician. In some cases, the expected delivery date was not available but the case narratives included a gestational age. In these cases, the conception date was adjusted according to the actual delivery date.

### Statistical analysis

The analyses included descriptive statistics of the pregnancies exposed to GH, GH exposure by country, baseline characteristics of exposed pregnancies, GHRT in exposed pregnancies, and outcomes in exposed and non-exposed pregnancies by age at conception. No statistical models were used for this analysis. There was no adjustment for missing data.

## Results

### Pregnancies exposed to GH

Overall, 54 pregnancies were reported in 40 female patients from the full analysis set of NordiNet® IOS (n = 20,195) and ANSWER (n = 20,813). A total of 47 pregnancies were exposed to GH between conception and delivery.

The complete exposure data were not available for five pregnancies; these were classified as having ‘unknown exposure’. The conception date was not available for two pregnancies, both of which ended in termination. Although these pregnancies were likely to have been exposed to GH at conception, the exact duration of exposure cannot be ascertained. During one pregnancy, treatment was continued until termination and is classified as ‘exposed up to termination’. For the other pregnancy, treatment was discontinued at an unknown time before termination and is classified as having ‘unknown exposure’.

The remaining seven of the overall 54 pregnancies were considered not exposed: GHRT was stopped 2 weeks or more prior to conception. Twenty-eight women had one pregnancy, 10 women had two, while two women had three pregnancies.

At conception, the median (range) age of patients with exposed pregnancies and available conception dates was 32.9 years (23.0–41.8). An exact age at conception was unavailable for two exposed pregnancies, as the conception dates were not available.

### Baseline characteristics of exposed pregnancies

**Fig. 1:**
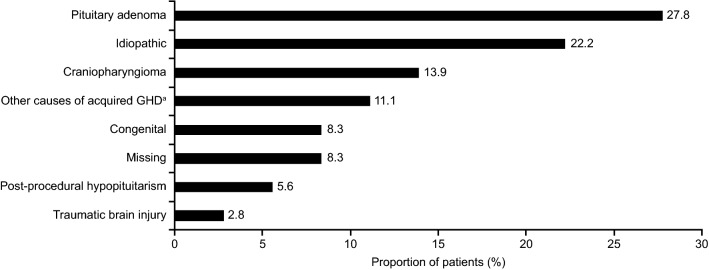
Etiology of pituitary diseases. ^a^Other causes of acquired GHD consisted of: GHD due to Sheehan syndrome, sarcoidosis, and unspecified GHD. *GHD* growth hormone deficiency

Baseline characteristics and etiologies of GHD of exposed pregnancies are summarized in Table 1 and Fig. 1, respectively. The most commonly reported pituitary-related conditions prior to conception were: hypopituitarism (21.3%), GHD owing to a pituitary tumor or its treatment (17.0%), diabetes insipidus (14.9%), craniopharyngioma (10.6%), and gonadotropin insufficiency (8.5%). The most commonly reported non-pituitary-related comorbidities prior to conception were: unspecified hyperlipidemia (4.3%), reduced vitality and energy (4.3%), acanthosis nigricans (2.1%), unspecified allergies (2.1%), unspecified asthma (2.1%), asymptomatic human immunodeficiency virus infection (2.1%), and autoimmune thyroiditis (2.1%).

**Table 1:** Baseline characteristics of exposed pregnancies (n = 47)

Variable	Level	n	%
IGF-I SDS before conception	Missing	15	31.9
	< − 2	4	8.5
	− 2 to 2	27	57.4
	> 2	1	2.1
Onset of pituitary disease	Adult onset	29	61.7
	Childhood onset	16	34.0
	Missing	2	4.3
Age at conception (years)^a^	Unknown	2	4.3
	< 30	9	19.1
	30–35	29	61.7
	> 35	7	14.9
BMI at conception (kg/m^2^)	Missing	14	29.8
	< 25	15	31.9
	25–35	16	34.0
	> 35	2	4.3
Pituitary hormone deficiency type	GH deficiency	41	87.2^b^
	Gonadotropin deficiency	24	51.1
	TSH deficiency	22	53.7
	ACTH deficiency	14	34.1
	ADH deficiency/diabetes insipidus	11	26.8
Pituitary deficiencies number	1 deficiency	11	26.8
	2 deficiencies	6	14.6
	3 deficiencies	13	31.7
	4 deficiencies	5	12.2
	5 deficiencies	6	14.6

*ACTH* adrenocorticotropic hormone, *ADH* antidiuretic hormone, *BMI* body mass index, *GH* growth hormone, *IGF-I* insulin-like growth factor-1, *SDS* standard deviation score, *TSH* thyroid-stimulating hormone

^a^Age at conception was calculated for 45 pregnancies exposed at conception

Conception dates were unavailable for two exposed pregnancies, which resulted in termination; however, it was still possible to determine the correct age category at conception for these pregnancies. As patients could have multiple pituitary deficiencies, the total number of pituitary deficiencies exceeded 47

^b^In six (12.8%) exposed pregnancies, GH deficiency was not listed as the primary diagnosis in the case report form

### GH exposure by country

Pregnancies were reported in 19 clinics across eight countries. Table 2 summarizes GH exposure across all stages of pregnancy by country in all 54 reported pregnancies.

**Table 2:** Growth hormone exposure during pregnancy by country in all 54 reported pregnancies

Country	Unknown exposure^a^	Not exposed	Exposed at conception	Growth hormone replacement stopped during the first trimester	Growth hormone replacement stopped during the second trimester	Growth hormone replacement stopped during the third trimester	Continued	Exposed up to termination^b^	Total
Belgium	0 (0.0)	0 (0.0)	0 (0.0)	1 (1.9)	0 (0.0)	0 (0.0)	0 (0.0)	0 (0.0)	1 (1.9)
Czech Republic	0 (0.0)	2 (3.7)	1 (1.9)	0 (0.0)	1 (1.9)	0 (0.0)	2 (3.7)	0 (0.0)	6 (11.1)
Denmark	4 (7.4)	2 (3.7)	1 (1.9)	2 (3.7)	6 (11.1)	0 (0.0)	7 (13.0)	1 (1.9)	23 (42.6)
France	0 (0.0)	1 (1.9)	1 (1.9)	1 (1.9)	0 (0.0)	0 (0.0)	1 (1.9)	0 (0.0)	4 (7.4)
Germany	1 (1.9)	2 (3.7)	1 (1.9)	7 (13.0)	1 (1.9)	0 (0.0)	1 (1.9)	0 (0.0)	13 (24.1)
Sweden	0 (0.0)	0 (0.0)	0 (0.0)	0 (0.0)	0 (0.0)	0 (0.0)	2 (3.7)	0 (0.0)	2 (3.7)
UK	0 (0.0)	0 (0.0)	0 (0.0)	1 (1.9)	0 (0.0)	0 (0.0)	0 (0.0)	0 (0.0)	1 (1.9)
USA	0 (0.0)	0 (0.0)	1 (1.9)	1 (1.9)	0 (0.0)	1 (1.9)	1 (1.9)	0 (0.0)	4 (7.4)
Total	5 (9.3)	7 (13.0)	5 (9.3)	13 (24.1)	8 (14.8)	1 (1.9)	14 (25.9)	1 (1.9)	54 (100.0)

Data are n (%)

Categories were defined as follows: ‘unknown exposure’: exposure cannot be determined due to missing pregnancy dates or missing exposure data; ‘not exposed’: pregnancies were not exposed at conception and beyond; ‘exposed at conception’: treatment was stopped between 2 weeks before and 2 weeks after conception; ‘growth hormone replacement stopped during the first trimester’: treatment was stopped between 2 weeks after conception and up to the end of the first trimester; ‘growth hormone replacement stopped during the second trimester’: treatment was stopped between the end of the first trimester and up to the end of second trimester; ‘growth hormone replacement stopped during the third trimester’: treatment was stopped between the end of the second trimester and up to 2 weeks prior to the end of the third trimester; ‘continued’: pregnancy was exposed beyond 2 weeks prior to the end of the third trimester; ‘exposed up to termination’: pregnancy was exposed up to termination but conception date was unavailable

^a,b^The conception date was not available for two pregnancies, both of which ended in termination. Although these pregnancies were likely to have been exposed at conception, the exact duration of exposure cannot be ascertained: ^a^for one pregnancy, treatment was stopped at an unknown time before termination and was classified as having ‘unknown exposure’; ^b^for the other pregnancy, treatment was continued until termination and was classified as ‘exposed up to termination’

### Growth hormone therapy in exposed pregnancies

**Fig. 2:**
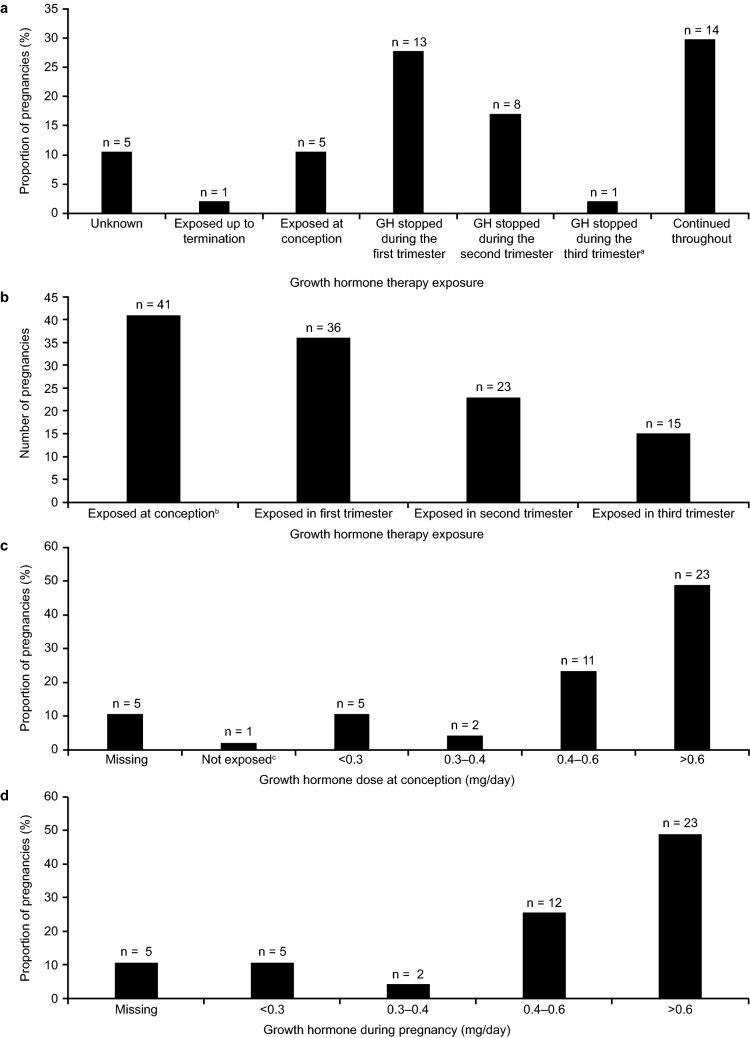
Growth hormone therapy in exposed pregnancies. **a** Growth hormone therapy during pregnancy. **b** Cumulative number of pregnancies exposed to growth hormone during pregnancy. **c** Growth hormone dose at conception. **d** Growth hormone dose during pregnancy. ^a^Until 14 days before delivery. ^b^The number of pregnancies cumulatively exposed at conception or with unknown exposure amounts to fewer than 47 because, in one pregnancy, growth hormone replacement therapy started in the first trimester. Exposure to growth hormone at conception, first trimester, second trimester, third trimester, or up to termination was defined as exposure before and after 2 weeks of each pregnancy stage (**b**). Growth hormone dose data were not available for five pregnancies, for which conception date was unknown (missing; **b**). ^c^Patients not exposed corresponds to one patient who started growth hormone replacement therapy after conception (**c**). The growth hormone dose of this patient during pregnancy is also included in **d**

**Fig. 3:**
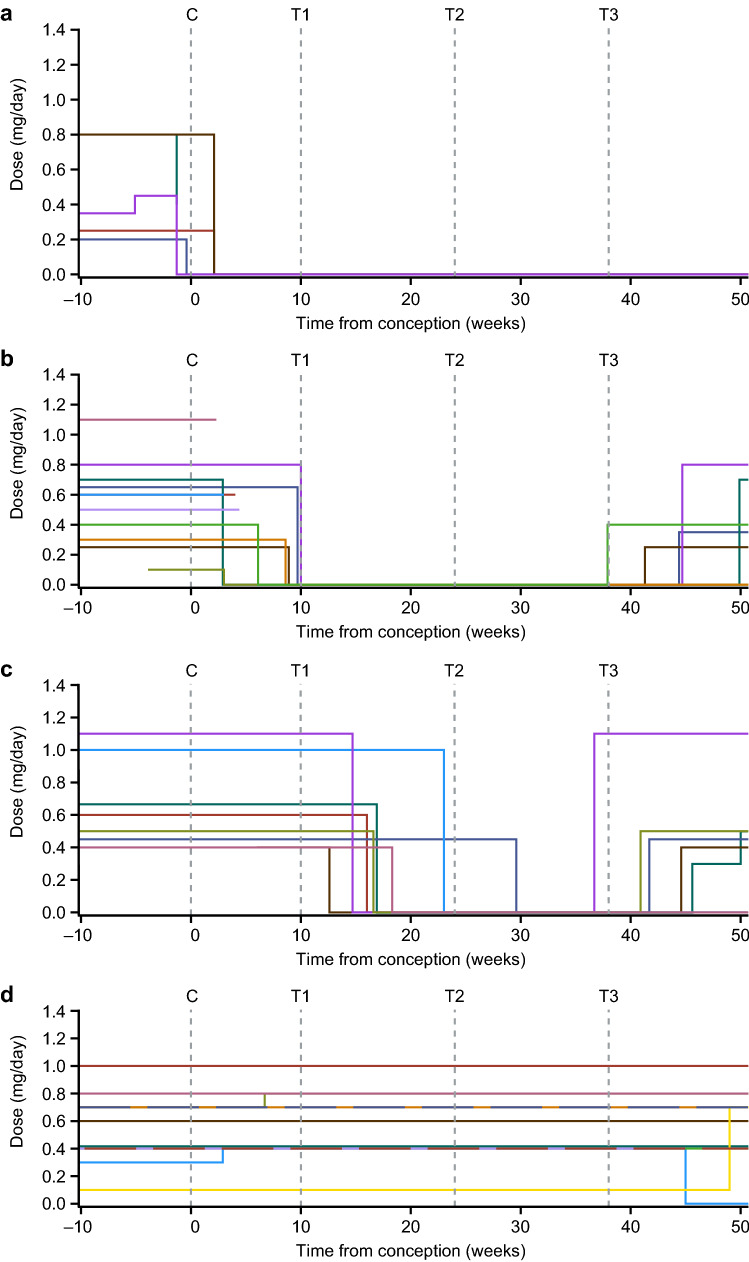
Growth hormone dose over time. **a** Pregnancies exposed to growth hormone only at conception. **b** Growth hormone replacement stopped during the first trimester. **c** Growth hormone replacement stopped during the second or third trimesters. **d** Pregnancies exposed to growth hormone throughout pregnancy. Spaghetti plots of each patient’s exposure to growth hormone throughout pregnancy. Each color line represents one patient. In **b** and **c**, lines that rise and then continue horizontally after the third trimester represent postpartum resumption of growth hormone replacement therapy. *C* conception, *T1* end of first trimester, *T2* end of second trimester, *T3* end of third trimester

Treatment with GH in exposed pregnancies is summarized in Fig. 2. The majority of pregnancies (n = 47; 87.0%) were exposed to GH (Fig. 2a). Five pregnancies (10.6%) were exposed at conception only. GH replacement therapy was continued after conception in 22 (46.8%) pregnancies, and then stopped during the first trimester in 13 pregnancies (27.7%), during the second trimester in eight pregnancies (17.0%), and during the third trimester in one pregnancy (2.1%). In 14 (29.8%) pregnancies (half of which were reported from Danish patients; Table 2), GH was continued throughout pregnancy. The cumulative proportion of pregnancies exposed to GH from conception to the third trimester is shown in Fig. 2b. In almost half of the pregnancies (48.9%), the GH dose was greater than 0.6 mg/day at conception (Fig. 2c). During the pregnancy, the GH dose was greater than 0.6 mg/day in just under half of the pregnancies (48.9%) (Fig. 2d). GH dose data were not available for five pregnancies, for which the conception date was unknown. Figure 3 shows spaghetti plots of the GH dose over time (before and after conception) for each patient exposed to GH, for whom data were available.

### Pregnancy outcomes

Pregnancy outcomes in exposed and non‑exposed pregnancies are shown in Table 3. Of the 47 pregnancies that were exposed to GH, 37 (78.7%) progressed to normal delivery. Thirty-one of the normal deliveries occurred at full term. One normal delivery occurred post-term, while two occurred at moderate-to-late pre-term. Gestational age was not available for the remaining three normal deliveries. Seven pregnancies ended in termination; five were due to spontaneous abortion (gestational age was available for three of these terminations: 12.6, 15.9, and 7.6 weeks). Out of the five pregnancies that ended as spontaneous abortions, two were exposed at conception and during the first trimester while, in another pregnancy, GHRT was continued and stopped during the second trimester. Duration of exposure was unknown for the other two cases. One termination resulted from a medical indication (no further information available), while the other was owing to the patient’s decision. For these two terminations, the exposure duration was unknown. No abnormalities were reported in the fetuses of pregnancies that progressed to spontaneous abortions. The pregnancy outcome was unknown in three cases.

**Table 3:** Outcomes in exposed and non-exposed pregnancies by age at conception

	Unknown	Delivery	Termination^a^	Spontaneous abortion	Total
Unknown age					
Exposed	0 (0.0)	0 (0.0)	1 (2.1)	1 (2.1)	2 (4.3)
Not exposed	0 (0.0)	0 (0.0)	0 (0.0)	0 (0.0)	0 (0.0)
< 30 years					
Exposed	1 (2.1)	8 (17.0)	0 (0.0)	0 (0.0)	9 (19.2)
Not exposed	0 (0.0)	4 (57.1)	0 (0.0)	0 (0.0)	4 (57.1)
30–35 years					
Exposed	2 (4.3)	24 (51.1)	0 (0.0)	3 (6.4)	29 (61.7)
Not exposed	0 (0.0)	3 (42.9)	0 (0.0)	0 (0.0)	3 (42.9)
> 35 years					
Exposed	0 (0.0)	5 (10.6)	1 (2.1)	1 (2.1)	7 (14.9)
Not exposed	0 (0.0)	0 (0.0)	0 (0.0)	0 (0.0)	0 (0.0)
Total					
Exposed	3 (6.4)	37 (78.7)	2 (4.3)	5 (10.6)	47 (100.0)
Not exposed	0 (0.0)	7 (100.0)	0 (0.0)	0 (0.0)	7 (100.0)

Data are n (%)

Exposure to growth hormone at conception, first trimester, second trimester, third trimester, or up to termination was defined as exposure within 2 weeks of each pregnancy stage

^a^One termination was the patient’s decision; the other termination was due to medical indication

All of the seven pregnancies not exposed to GH (for which GHRT was stopped more than 2 weeks prior to conception and/or initiated after delivery/termination) progressed to normal delivery. Six of these occurred at full term. The gestational age was unknown for the remaining delivery.

There were three AEs reported in two pregnancies in two different patients who were exposed to GH; in one pregnancy, pertaining to a 32-year-old patient who was treated with GH until 25 days after conception, it was reported that the baby did not drop and that induction of labor failed. As a result, the patient had a non-elective caesarean section. The patient delivered a child at 40 weeks and 4 days. The delivery was a live birth with no congenital anomalies, malformations, or health problems. The newborn child was a male, with a length of 51 cm (20 inches; − 0.2 standard deviation score [SDS]), and weight of 2.954 kg (6 lb and 8 oz; − 1.2 SDS). The newborn had an Apgar score of 8 and 9. The physical examination of the newborn was normal. The third AE was reported in a 31-year-old patient who was treated with GH until the 20th week of pregnancy. At 37 weeks of gestation, the patient experienced pre-eclampsia. The patient requested a caesarean section, delivering a live female baby. The Apgar score was reported as 10 at 5 min after birth. There was no obstetrical complication reported during delivery. The newborn had no illness or abnormalities. These three AEs were assessed to be unlikely to be related to GH treatment.

## Discussion

This analysis of pregnancy data and outcomes from two large non-interventional, real-world studies, NordiNet® IOS and the ANSWER Program, combining data from 47 pregnancies exposed to GH during pregnancy, provides insight into the use of GH replacement during pregnancy in real-world practice in Europe and the USA.

It is notable that, in NordiNet® IOS and the ANSWER Program, GHRT was at least partially continued at a constant dose during pregnancy for many women who became pregnant. Almost 79% of exposed pregnancies resulted in a normal delivery. Neonatal complications were not reported for this study. The observed termination rate (14.9%) was consistent with data from a study in Denmark that reported a 13.5% spontaneous abortion rate in the general population [23]. However, spontaneous abortions could have been under-reported owing to denial, forgetfulness, misattribution as delayed menstruation [24], or because of a long delay between termination and the next clinical appointment with the endocrinologist. Despite limitations relating to missing information and potential under-reporting, data from NordiNet® IOS and ANSWER corroborate findings from previous reports [12, 13, 23, 25], and provide additional real-world data about the safety profile of GHRT in pregnant adults with GHD. Three AEs were reported in two pregnancies, which were assessed to be unlikely to be related to the GHRT.

At the time of pregnancy, three different practices regarding GHRT were observed: discontinuation of the hormone replacement therapy, continuation of GH throughout pregnancy, and discontinuation of GHRT sometime during the first or second trimester. During pregnancy, pituitary GH decreases in the second trimester [11]. Vila and colleagues suggest that gradually reducing GHRT to a complete cessation during the second trimester could better mimic normal physiology in pregnant women with GHD [14]. As pregnancies in these patients are rare, the question of whether and when to discontinue GH replacement is a key clinical issue in this patient population. There may be concerns about continuing GH replacement for too long or discontinuing too early, as most women with GHD can experience difficulty becoming pregnant and could have an increased risk of abortion.

This study has numerous strengths. NordiNet® IOS and ANSWER were not constrained by a highly specific protocol (as clinical trials are), and so offer an inclusive picture of the use and effectiveness of GHRT in clinical practice. Thus, these studies provide real-world evidence of the outcomes of GH use in pregnant women.

There are several potential limitations/biases in the current analysis. As in all non-interventional observational studies, the lack of an untreated control group limits the potential of drawing conclusions from the results. There could also be a potential confounding of the results by local differences in diagnostics, laboratory analyses, and reporting of events among different clinics and countries. Furthermore, data on potential antenatal complications like gestational diabetes and hypertension (which are recognized to have increased prevalence in the acromegaly population) were not available. Thus, it was not possible to ascertain the potential impact of these complications on the outcomes reported in this study, if any GHD patients were overtreated with replacement GH.

Overall, no safety signals were observed in relation to GH exposure in pregnant women with GHD. Additionally, these data are consistent with findings from previous studies reporting data in pregnancies exposed to GH, at conception or throughout pregnancy [12, 13, 23, 25]. GHRT is not approved for use during pregnancy and current guidelines recommend stopping GHRT after conception [26]. This study and others have shown satisfactory pregnancy outcomes following the decision of patients and physicians to continue GHRT at least into the second trimester, during which time pituitary GH secretion would begin a progressive decline to undetectable levels during the second half of pregnancy while placental GH is rising. Prospective studies assessing the effect of GHRT during pregnancy on both maternal and child outcomes are needed.

## Data Availability

The study protocol and redacted clinical study reports are available according to Novo Nordisk data sharing commitments. Individual participant data have been shared with the study investigators in a pseudonymized format. The data will be available as redacted study reports permanently after research completion and approval of product use in both the EU and USA with no end date. Data will be shared with bona fide researchers submitting a research proposal requesting access to data, for analyses in line with the aims of the study protocol and approved by the Independent Review Board according to the IRB charter (see novonordisk-trials.com). Request proposal forms and the access criteria can be found at novonordisk-trials.com. The data will be made available on a specialized SAS data platform.
